# Clinical decision-making in older adults following emergency admission to hospital. Derivation and validation of a risk stratification score: OPERA

**DOI:** 10.1371/journal.pone.0248477

**Published:** 2021-03-18

**Authors:** Khushal Arjan, Lui G. Forni, Richard M. Venn, David Hunt, Luke Eliot Hodgson

**Affiliations:** 1 Brighton and Sussex Medical School, Brighton, United Kingdom; 2 Department of Clinical & Experimental Medicine, Faculty of Health Sciences, University of Surrey, Guildford, United Kingdom; 3 Intensive Care Unit, Royal Surrey Hospital, Guildford, Surrey, United Kingdom; 4 Department of Medicine for the Elderly and Intensive Care, Worthing Hospital, Western Sussex Hospitals NHS Foundation Trust, Worthing, United Kingdom; 5 Intensive Care, Worthing Hospital, Western Sussex Hospitals NHS Foundation Trust, Worthing, United Kingdom; Indiana University School of Medicine, UNITED STATES

## Abstract

**Objectives of the study:**

Demographic changes alongside medical advances have resulted in older adults accounting for an increasing proportion of emergency hospital admissions. Current measures of illness severity, limited to physiological parameters, have shortcomings in this cohort, partly due to patient complexity. This study aimed to derive and validate a risk score for acutely unwell older adults which may enhance risk stratification and support clinical decision-making.

**Methods:**

Data was collected from emergency admissions in patients ≥65 years from two UK general hospitals (April 2017- April 2018). Variables underwent regression analysis for in-hospital mortality and independent predictors were used to create a risk score. Performance was assessed on external validation. Secondary outcomes included seven-day mortality and extended hospital stay.

**Results:**

Derivation (n = 8,974) and validation (n = 8,391) cohorts were analysed. The model included the National Early Warning Score 2 (NEWS2), clinical frailty scale (CFS), acute kidney injury, age, sex, and Malnutrition Universal Screening Tool. For mortality, area under the curve for the model was 0.79 (95% CI 0.78–0.80), superior to NEWS2 0.65 (0.62–0.67) and CFS 0.76 (0.74–0.77) (P<0.0001). Risk groups predicted prolonged hospital stay: the highest risk group had an odds ratio of 9.7 (5.8–16.1) to stay >30 days.

**Conclusions:**

Our simple validated model (Older Persons’ Emergency Risk Assessment [OPERA] score) predicts in-hospital mortality and prolonged length of stay and could be easily integrated into electronic hospital systems, enabling automatic digital generation of risk stratification within hours of admission. Future studies may validate the OPERA score in external populations and consider an impact analysis.

## Introduction

In the United kingdom (UK) the over 65 years demographic have demonstrated the largest increase in emergency hospital admissions of any age group, a trend projected to continue further in the coming decade [1]. Care pathways for this cohort must not only optimise clinical management but also involve patients and their kin in decision-making with regard to escalation and limitations of care. This should be aided by objective measures, including where appropriate, accurate prognostication [2]. A generalisable prediction score developed for acutely unwell older patients may help develop and inform such pathways.

In routine UK acute clinical practice, methods to monitor and determine escalations of care are limited to the National Early Warning Score (NEWS) and its iteration NEWS2 [3], which combines physiological parameters into a risk score, and clinical judgement. However, NEWS has demonstrated only moderate predictive power for mortality in older cohorts [4, 5].

Frailty has been investigated as a method to risk assess this demographic [6]. In UK hospital settings, frailty is commonly quantified using the Clinical Frailty Scale [7]. This tool assesses domains such as co-morbidity, function, and cognition to create a score ranging from very fit to terminal illness. Risk prediction models incorporating frailty are yet to be implemented in the acute clinical setting for older patients.

Clinical frailty, acute physiology (NEWS2), and other variables such as nutrition assessments, blood parameters, previously coded past medical history, and drugs prescriptions, are being increasingly employed in electronic systems which may themselves facilitate enhanced clinical risk stratification [8, 9]. This study aimed to derive and validate a prediction score for in-hospital mortality in the older adult patient presenting to hospital, using readily accessible electronic information, that would allow subsequent digital automation and presentation to the clinical team.

## Materials and methods

### Source of data and participants

Data was collected prospectively for all patients (≥65 years) admitted through two emergency departments (EDs) of a non-specialist hospital organisation on the South coast of England that has 870 beds and a combined annual ED attendance over 135,000 (April 2017 –April 2018). The two hospital sites, with minimal overlap of staffing were treated as independent populations to derive (site 1) and validate (site 2) the model. Participants were excluded if: no NEWS2 or clinical frailty scale (CFS) was recorded, or the length of stay in hospital was <1 day, to limit non-urgent attendances.

### Outcome

The primary outcome was in-hospital mortality. Secondary outcomes were: 48-hour mortality, 7-day mortality, hospital stay >30 days, and re-admission <30 days of discharge.

### Predictors

Variables collected included: age, sex, co-morbidity (ICD-10 [10]) coding of congestive cardiac failure, diabetes, liver disease, chronic kidney disease (eGFR <60mls/min), community-acquired acute kidney injury (CA-AKI) [11], NEWS2, clinical frailty scale (CFS), Malnutrition Universal Screening Tool (MUST) [12] and palliative care or a do not attempt resuscitation (DNAR) status recorded. Definitions and explanations of the variables measured are presented in Appendix 1 in S1 File. These variables are routinely collected for older patients at both sites and are known to be used in other scoring systems.

### Missing data

The missingness of NEWS2 and CFS, in patients who did not have it documented, was assessed for correlation with in-hospital mortality. Any remaining missing data, after the exclusion of patients without a documented NEWS2 and CFS, was handled with multiple imputation, using the most appropriate method [13].

### Statistical analysis

Binary logistic regression was carried out using a backwards stepwise elimination method on accessible and clinically significant variables. The identified independent predictors of in-hospital mortality were used to create a risk prediction score. Model performance was assessed for calibration with calibration plot analysis and Hosmer-Lemeshow test and area under receiver operating curve (AUC) for discrimination, and R^2^ for determination (Cox-Snell). Re-calibration and re-adjustment of the model were undertaken to optimise the risk score in the external validation group [14]. A scoring system was created by assigning points to the independent predictors of in-hospital mortality. Points were allocated by dividing their regression coefficients (β), by the smallest β of all variables. These numbers were then rounded to the nearest integer [15]. A step-by-step example of points allocation is provided in Appendix 2 in S1 File.

Information loss between the models, when using regression coefficients or the points score, was assessed by differences in AUC [16]. Sensitivity analysis on the full model examined performance differences when excluding patients under palliative care or with a DNAR order in-place.

### Risk groups

Low, medium, high and severe risk groups were created using cut-offs that optimised the sensitivities, specificities, and mortality risk for use in clinical practice.

### Development and validation

Both hospitals are within the same NHS England Trust. There were no systematic differences in the method of data collection or admission criteria between derivation and validation datasets.

Data was analysed using IBM SPSS Statistics version 25^®^ and Stata version 15.1^®^.

### Reporting guidelines

This study remained concordant with the TRIPOD checklist for predictive modelling [13].

### Ethical approval

Ethical approval was given by NHS South Central—Hampshire B Research Ethics Committee (REC reference 18/SC/0513).

## Results

### Participants characteristics

14,274 non-elective non-day case admissions aged ≥65 years, were admitted in site 1 and 14,951 in site 2, during the study period. 5,300 and 6,560 were excluded from site 1 and 2 respectively, for missing either NEWS2 or CFS, leaving 8,974 and 8,391 patients to be included for analysis in the derivation and validation groups respectively (consort chart presented in Appendix 3 in S1 File). Clinical, demographic, and missing data for the derivation and validation sites are presented in Table 1. Univariate analysis for in-hospital mortality is presented in Appendix 4 in S1 File.

**Table 1:** Baseline characteristics for each site.

	Derivation cohort (n = 8,974)	Validation cohort (n = 8,391)	Overall (n = 17,365)	P value
**Demographics**				
Male (%)	4088 (45.6)	3856 (46.0)	7944 (45.7)	0.607^a^
Median age (IQR)	84 (77–90)	82 (76–88)	82 (74–88)	<0.001^b^
**Clinical variables n (%)**				
Congestive cardiac failure	1,061 (11.8)	862 (10.3)	1,923 (11.1)	0.001^a^
Chronic kidney disease	3,988 (44.4)	3,430 (40.9)	7,418 (42.7)	<0.001^a^
Liver disease	61 (0.7)	23 (0.3)	84 (0.5)	<0.001^a^
Diabetes	1,342 (15.0)	1,216 (14.5)	2,558 (14.7)	0.415^a^
Palliative care or DNAR	1,250 (13.9)	897 (10.7)	2,147 (12.4)	<0.001^a^
CA-AKI	936 (10.4)	787 (9.4)	1,723 (9.9)	0.023^a^
**Clinical scores**				
Median MUST (IQR)	1 (1–2)	1 (1–2)	1 (1–2)	<0.001^b^
Median NEWS2 (IQR)	1 (0–3)	1 (0–3)	1 (0–2)	0.001^b^
Mean CFS (SD)	5.0+/-1.69	4.9+/-1.66	5.0 +/-1.68	<0.001^c^
**Outcomes**				
Median days in-hospital (IQR)	10 (4–21)	9 (5–16)	9 (5–18)	<0.001^d^
Length of stay >30 days (%)	1,191 (13.3)	582 (6.9)	1,773 (10.2)	<0.001^a^
30-day readmission (%)	1,773 (19.8)	1,681 (20.0)	3,454 (19.9)	0.662^a^
Death (%)	928 (10.3)	610 (7.3)	1,538 (8.9)	<0.001^a^
**Missing data (excluded)**				
Missing only NEWS2 (%)	225 (4.2)	152 (2.3)		
Missing only CFS (%)	4828 (91.1)	6224 (94.9)		
Missing both NEWS2 & CFS (%)	217 (4.1)	183 (2.8)		
Total missing	5,300	6,560		

^a^ Pearson Chi squared

^b^ Mann Whitney U Rank Sum Test

^c^ Welch’s T-Test.

Median and IQR for NEWS2 and MUST are the same between sites.

Statistical significance for difference demonstrates a difference in the spread of data, measured by the Mann Whitney U rank test.

CA-AKI—Community-acquired acute kidney injury, CFS—clinical frailty scale,

DNAR—do not attempt resuscitation order,

IQR—interquartile range, MUST—malnutrition universal screening tool,

NEWS2 –national early warning score 2.

### Missing data

358 (4.0%) cases had missing MUST scores in the derivation cohort and 105 (1.3%) in the validation. The Markov Chain Monte Carlo method of multiple imputation was employed (7,020 values were imputed for 468 missing items over 15 iterations) [17]. Independent predictors identified were not different between the original data and after multiple imputation.

### Model specification

Regression analysis identified six independent predictors of mortality: male sex, age, CA-AKI, MUST, NEWS2, and CFS (Table 2), with no evidence of collinearity (Appendix 5 in S1 File).

**Table 2:** Independent predictors of in-hospital mortality and points allocation.

Variable	Regression coefficient (β)	p value	Odds ratio	Formula	Points allocation
CA-AKI	0.809	<0.001	2.25 (1.86–2.71)	0.809÷0.200≈	4
CFS	0.345	<0.001	1.41 (1.34–1.48)	0.345÷0.200≈	2
MUST per increase in risk group	0.284	<0.001	1.33 (1.22–1.45)	0.284÷0.200≈	1
NEWS2	0.229	<0.001	1.26 (1.22–1.29)	0.220÷0.200≈	1
Age	0.04	<0.001	1.04 (1.03–1.05)		N/A
Age (every 5 years over 65)	0.2			0.200÷0.200≈	1
Male	0.321	<0.001	1.38 (1.19–1.60)	0.321÷0.200≈	2
Constant	-8.452	<0.001	0		

CA-AKI—Community-acquired acute kidney injury, MUST—malnutrition universal screening tool, NEWS2 –national early warning score 2.

A points-based score was created using these independent predictors and for ease of use was capped at 50 with the model abbreviated to the Older Persons’ Emergency Risk Assessment (OPERA) score. For the MUST variable, one point is added for every increase in risk group (low 0, medium 1, and high risk 2 points, respectively).

### Model performance

**Fig 1:**
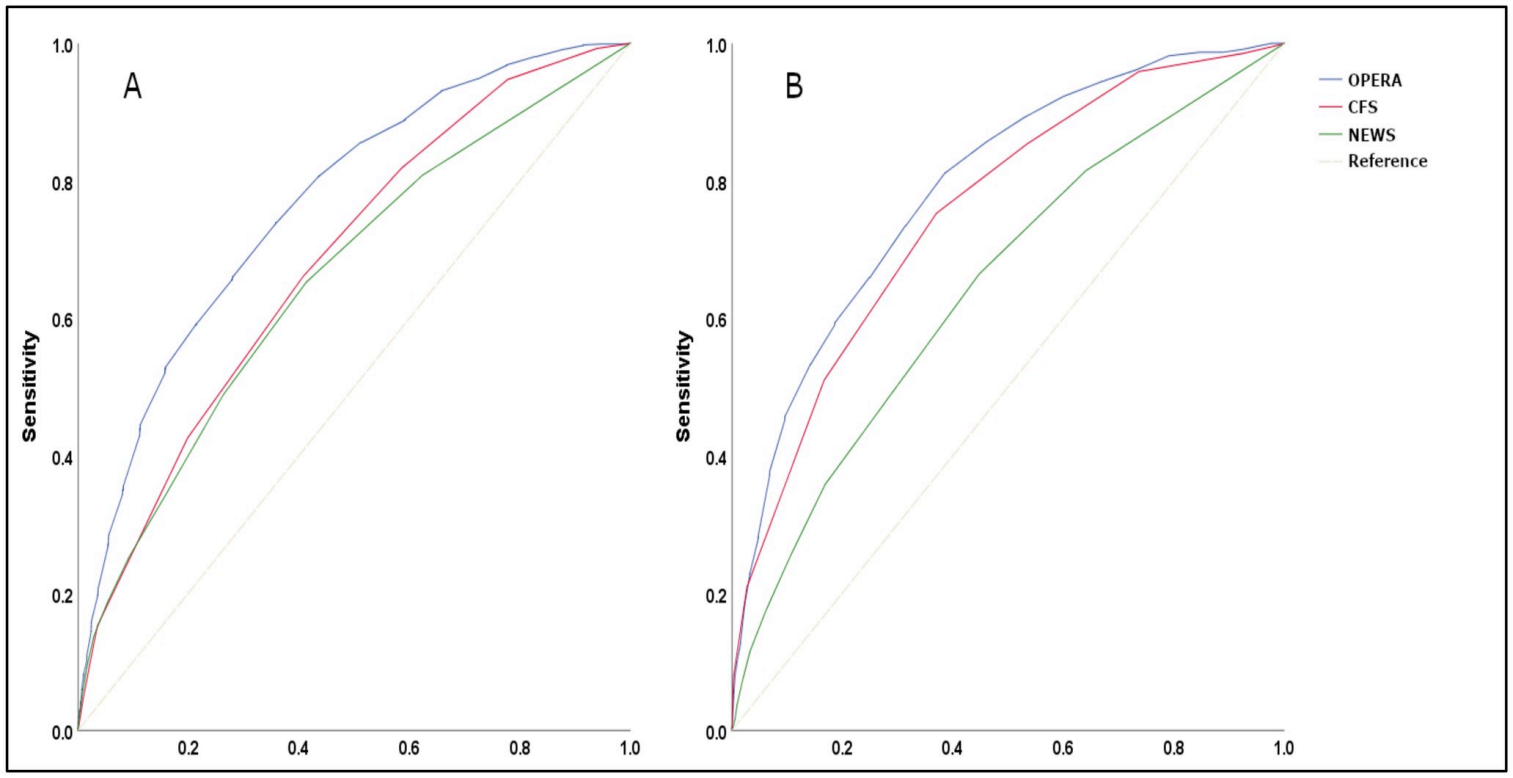
Receiver operating curves for in-hospital mortality for derivation and validation groups. (A) derivation and (B) validation groups. OPERA score (Blue line), CFS (Red line) and NEWS2 (Green line). CFS—clinical frailty scale, NEWS2—national early warning score.

There was no statistically significant difference in AUC for mortality prediction when using the regression coefficients or OPERA points score (p = 1), indicating negligible information loss. Likewise, R^2^ and Hosmer-Lemeshow tests demonstrated negligible difference. For in-hospital mortality, the derivation model demonstrated an R^2^ of 0.087 and AUC of 0.77 (95% confidence interval 0.75–0.78) (Fig 1). The score in the validation group had an R^2^ of 0.082 and an AUC of 0.79 (0.78–0.80). The model’s calibration in the validation cohort as measured by the Hosmer-Lemeshow test was acceptable (p = 0.302). Excluding the variable requiring a laboratory result (creatinine to define CA-AKI), AUC was lowered to 0.76 (0.74–0.77) in the derivation cohort and 0.77 (0.76–0.79) in the validation cohort.

AUC for NEWS2 was 0.66 (0.64–0.68) and 0.65 (0.62–0.67) in derivation and validation sets, respectively. AUC for CFS were 0.68 (0.66–0.70) and 0.76 (0.74–0.77) in the derivation and validation set, respectively. Pairwise comparisons for in-hospital mortality AUC were significantly different between OPERA and for NEWS2 and CFS (p<0.0001) [16].

OPERA was used to classify individuals into low, medium, high, and severe risk groups (sensitivity, specificity, positive and negative predictive values are presented for each risk group in Appendix 6 in S1 File). For the validation set negative predictive value (NPV) was greater than 95% for each risk group.

In derivation, AUCs to predict secondary outcomes were: 48-hour mortality 0.86 (0.83–0.90), 7-day mortality 0.83 (0.81–0.85), hospital stay >30 days 0.66 (0.64–0.67), and 30-day re-admission 0.50 (0.49–0.52). In the validation cohort AUCs for the same outcomes were: 0.84 (0.78–0.90), 0.83 (0.79–0.86), 0.68 (0.65–0.70), and 0.52 (0.50–0.54), respectively (receiver operating curves for secondary outcomes are presented in Appendix 7 in S1 File). OPERA risk groups were investigated as indicators of extended hospital stay (>30 days) with odds ratios (ORs). When compared to the low risk group, the medium, high, and severe groups demonstrated increased ORs of hospital stay >30 days: 3.7 (95% CI 2.9–4.8), 7.9 (4.8–13.1), and 9.7 (5.8–16.1), respectively (ORs for hospital stay >7 days and >48 hours in derivation and validation sets are presented in Appendix 6 in S1 File).

### Sensitivity analysis

OPERA demonstrated higher discrimination for in-hospital mortality when excluding patients with limitations in care in place or with palliative care input: AUC 0.79 (0.76–0.82) in derivation and 0.80 (0.76–0.83) in validation. In derivation AUCs for mortality in those >75 years and >85 were 0.75 (0.73–0.77) and 0.74 (0.71–0.76), respectively; on the validation set, AUCs were 0.78 (0.76–0.80) and 0.75 (0.73–0.78), respectively.

**Fig 2:**
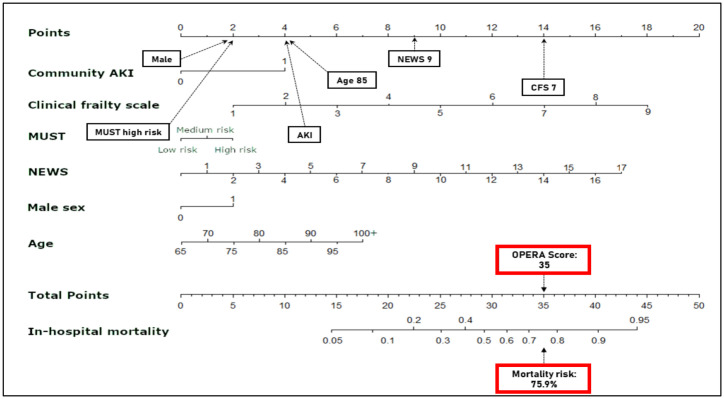
Nomogram of derivation model. Example shown is of an acutely admitted 85-year-old male with a CFS of 7, creatinine 163 μmol/L (baseline 80), high risk MUST, and a NEWS2 of 9. Age 85 = 4 points (one point added for every 5 years above 65), CFS 7 = 14 points (individual CFS multiplied by two), AKI = 4 points, high risk MUST = 2 points (low = 0, medium = 1 and high = 2), male sex = 2 points, NEWS2 of 9 = 9 points (one point for every NEWS2 score). Total OPERA score 35 points. AKI—acute kidney injury, MUST—Malnutrition Universal Screening Tool.

A nomogram based on the derivation risk equation is presented in Fig 2, with a worked example of an 85-year old man, an admission NEWS2 of 9, CFS of 7, high-risk on the MUST assessment and community-acquired AKI, giving an OPERA score of 35 with an estimated in-patient mortality of 75.9%. A further worked example using the regression equation and a graphical representation of mortality risk versus points scored are in the S1 File (Appendixes 8 and 9 in S1 File, respectively).

$$Mortality\;risk=(\frac{1}{1+e^{-(-8.452+65(0.04)+\;0.2(35))}})=0.759\;or\;75.9\%$$

An age constant “65(0.04)” within the equation allows for OPERA points only to be given for age past 65, as opposed to adding points from starting from age zero.

### Model updating

**Fig 3:**
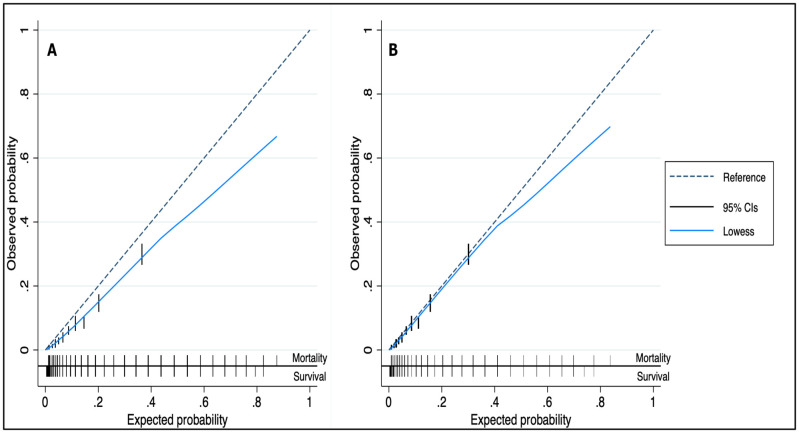
Calibration curve on validation cohort of OPERA points model before and after recalibration. (A) before recalibration, (B) after recalibration. Reference line, 95% confidence interval, lowess smoothing curve, and the distribution of mortality and survival against predicted probabilities.

Calibration plot analysis on the validation cohort demonstrated a small, statistically significant over-estimation of mortality risk (intercept difference -0.310) (Fig 3). The model was successfully re-calibrated through intercept updating (Appendix 10 in S1 File).

## Discussion

This study derived and externally validated the Older Persons’ Emergency Risk Assessment (OPERA) score, using routinely collected demographic and clinical information. OPERA could offer clinicians an objective method to help risk stratify for early risk of death and prolonged hospital stay and inform discussions with patients and families about potential escalation of management or limitations of care. The model’s high NPV could be particularly useful in identifying those truly low risk, unlikely to require care escalation. Two large and geographically separated populations presenting non-specifically to ED were used to derive and validate OPERA. This supports potential generalisability and reduces the likelihood of over-optimism, though further external validation is desirable. Model over-fitting was limited by assessing a succinct number of clinical variables.

The model demonstrated good discrimination in both cohorts. Calibration plot analysis revealed a small over-prediction of mortality risk in the validation set however this was addressed with simple re-calibration techniques. The initial disparity between predicted and observed mortality may be a result of the significant differences in demographic and medical presentations between the hospitals. Future studies may investigate these differences between both hospitals. Controlling mortality predictions for these previously unaccounted variables may help elucidate the cause of the disparity. Additionally, the regression method used, created a linear coefficient of risk for each variable; some variables may have non-linear associations with mortality. This can be integrated into scoring systems, however it increases complexity and makes it harder to use in a busy clinical setting.

Excluding those without a documented frailty score limited sample size. Within both hospitals, all patients over the age of 65 are expected to have a CFS assessment, however, this may not have been judged necessary by hospital staff and hence not done if the patient was relatively well. Further analysis supports this, as a documented CFS was independently associated with increased age, NEWS2, MUST, palliative care input, a do not attempt resuscitation (DNAR) form, and all co-morbidities. This may explain why having a documented frailty score was associated with an increased odds ratio for in-hospital mortality (Appendix 11 in S1 File). A key potential benefit of the OPERA model however, is to aid mortality prediction, and those with a complete CFS assessment are more likely to form part of this at-risk group for whom such risk stratification is important. Importantly, OPERA revealed better discrimination when excluding patients with a DNAR form in-situ or receiving palliative care. This group have already had decisions made on appropriate care and as such, it is the remaining cohort for whom such a score may be of most clinical use. Individuals without documented NEWS2 were also excluded. It is suspected the majority of patients missing NEWS2 were medically fit patients, either admitted to ED in order to access other services such as dialysis in the renal unit or, admitted overnight to await a home package of care. As such, these would not be the demographic that would require risk stratification. Other causes for missing NEWS2 scores may include self-discharge prior to triage, declining physical observations, death in-between entering ED and triage, or data not being entered in the electronic notes system.

OPERA performed better for short-term mortality, likely due to the function of NEWS2 to quantify acute illness severity. The ability of the model to predict longer-term outcomes requires further investigation. OPERA provided odds ratios for extended hospital stay depending on the patient’s risk category, which could be useful for clinicians and hospital management as it provides an objective risk estimate, that could support discharge planning and resource allocation. Importantly however, the confidence intervals produced for each risk group overlapped. This may be due to the lower incidence of patients experiencing a length of stay over 30 days thus increasing uncertainty over these results.

OPERA did not discriminate for 30-day re-admission, aligning with previous research demonstrating the limitation of physiological data in predicting this outcome [18]. Alternatively, more historical variables such as the number of previous admissions and adverse events post-admission may do better [19, 20]. The most similar variable in this regard is frailty, however it too struggles to predict longer term out-of-hospital outcomes [21]. Future studies could derive prediction models focusing on broader holistic and patient centred outcomes, which could also include quality of life following discharge and likelihood of discharge to long-term institutional care.

Although still performing well, discrimination was slightly lower when the model was applied only to patients >85; at such extremes of age accurate prognostication will remain challenging. Few other tools have been described in this field. The Risk Index for Geriatric Acute Medical Admission (RIGAMA) has similar aims of this study, with similar discrimination in validation [22]. RIGAMA aimed to quantify frailty and mortality risk based on the index of accumulated deficits, where the number of deficits contribute to individual risk, regardless of what those deficits are [23]. OPERA uses conventional modelling methods where each variable contributes its own amount of risk and unlike RIGAMA, does not require a working diagnosis and the results for bloods tests indicated only for certain diagnoses. Soong et al., reported models using frailty with AUCs ranging 0.62–0.66 [24], and Gilbert et al., reported a frailty risk score from ICD-10 diagnostic codes with a C-statistic of 0.60 [25].

### Clinical use and future implications

The ability of OPERA to predict in-hospital mortality makes it potentially applicable for use in older patients presenting acutely to hospital, for instance at the point of a post-take ward round. The score uses routinely collected data and is simple to use. Both MUST and the CFS can be quickly obtained by staff [26, 27], with the latter also demonstrating acceptable inter-rater agreement [7]. Additionally, NEWS2 is widely adopted in multiple UK healthcare settings [3]. Importantly, this data can be easily accessed from electronic patient record systems and calculated within hours of admission.

OPERA may be beneficial for junior staff and nurses, who make-up the majority of out-of-hours staffing, providing objective short-term prognostication and prompting potential need for care escalation or the opinion of a senior decision-maker. Furthermore, objective risk scores can inform difficult discussions with both patients and families by providing quantifiable population estimates in addition to an individual clinical assessment, rather than replacing clinical acumen. OPERA could also contribute to the early identification of palliative care needs which in turn may avoid inappropriate intensive treatment and help deliver appropriately holistic care [28]. As all variables were imported from the hospital’s electronic system, future services could automate elements of care recommendations, or provide real-time prompts to healthcare providers; computer based prediction tools can save time and limit mistakes compared to those limited to pen and paper [29]. Lastly, classification of patients into low, medium, high, and severe risk could be used to help determine care pathways. The utility of OPERA in acute older patient care could be further investigated with decision curve analysis [30] and impact studies [31].

## Conclusion

This study derived and validated OPERA, a risk score that could be digitally integrated into currently available healthcare electronic hospital systems at the point of hospital admission. Future research could validate OPERA in external populations and consider impact analysis.

## Supporting information

S1 File (DOCX)

S1 Data (XLSX)
