# Supplementary material for: Clinical decision-making in older adults following emergency admission to hospital. Derivation and validation of a risk stratification score: OPERA
Source: PLoS One. 2021 Mar 18;16(3):e0248477. doi: 10.1371/journal.pone.0248477 (PMC7971558; doi:10.1371/journal.pone.0248477)
Supplement: S1 File — (DOCX) [file pone.0248477.s001.docx]

S1 File

# Appendix 1: Definitions and explanations of variables measured

Frailty was measured using the Clinical Frailty Scale (CFS; ranging from 1-11) [1]. The hospitals use the Malnutrition Universal Screening Tool (MUST) which is then classified into low, medium, and high risk[2]; our score uses these risk categories and not the score itself. The National Early Warning Score (NEWS) is an acute illness severity score used for adults of all age (ranging from 0-17; low to high risk) [3]. Chronic kidney disease (CKD) was defined as a documented estimated glomerular filtration rate <60ml/min within the previous year. Community acquired acute kidney injury (CA-AKI) was defined using KDIGO [4] definitions of a rise in serum creatinine x1.5 baseline and the nationally accepted algorithm [5].

# Appendix 2: Example of points allocation methodology

If a diagnosis of congestive cardiac failure, CA-AKI, and male sex were identified as the independent predictors of in-hospital mortality, with beta-coefficients (β) of 0.83, 0.41, and 0.20 respectively, we would divide each β by the β for male sex (0.20), as this is the smallest. This would then be rounded to 1 significant figure.

Congestive cardiac failure 0.83 $\div$ 0.20 = 4.15 ~ 4 points

CA-AKI 0.41 $\div$ 0.20 = 2.05 ~ 2 points

Male sex 0.20 $\div$ 0.20 = 1 ~ 1 point

In this example, after rounding to the nearest whole number, our scoring system would allocate 4 points for congestive cardiac failure, 2 points for CA-AKI, and 1 point for being male; 7 points in total.

Thus, a female patient with congestive cardiac failure and CA-AKI would have 6 points out of a possible 7.

# Appendix 3: Patient flow chart


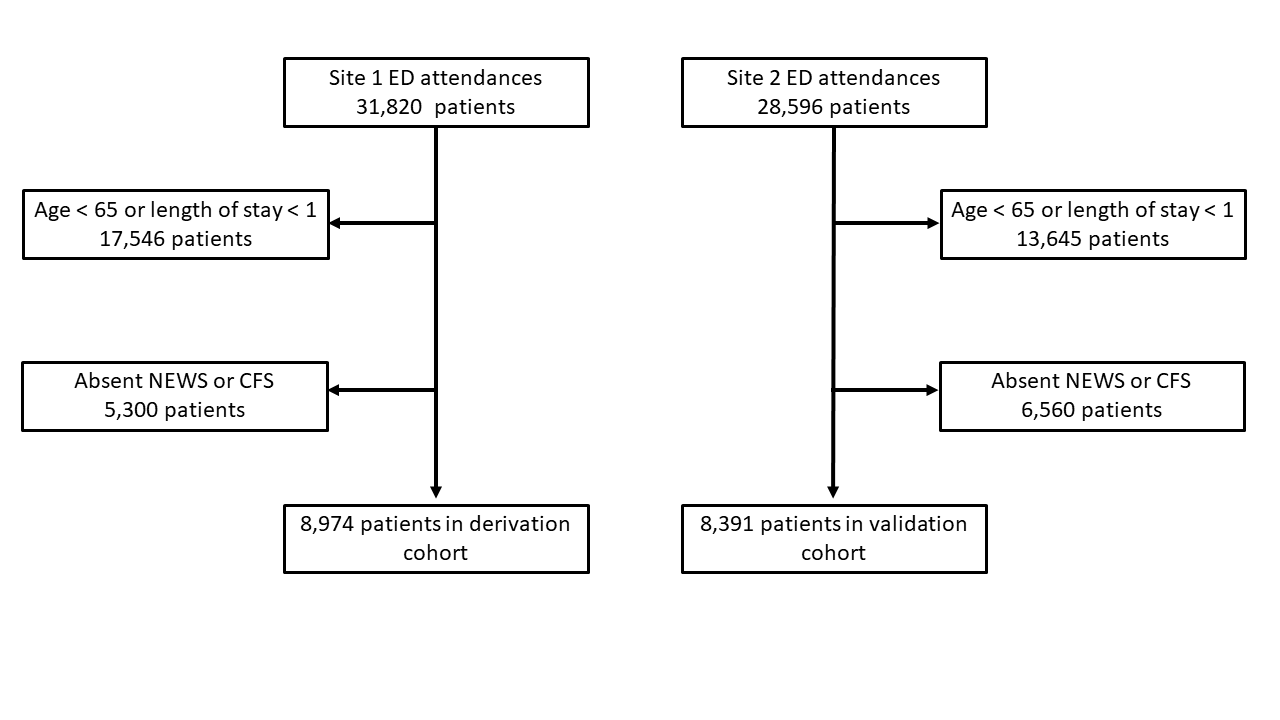


**Figure.** Flow chart of study participants.

# Appendix 4: Univariate analyses

**Table.** Univariate analysis for mortality in derivation and validation cohorts. Pearson Chi squared (^a^), Mann Whitney U Test (^b^) Welch's T-Test (^c^).

|  | **Derivation cohort** | | | **Validation cohort** | | |
| --- | --- | --- | --- | --- | --- | --- |
|  | Mortality  (n=928) | Survival  (n=8046) | P value | Mortality  (n=610) | Survival  (n=7781) | P value |
| **Demographics** |  |  |  |  |  |  |
| Male (%) | 454 (48.9) | 3634 (45.2) | 0.030^a^ | 294 (48.2) | 3562 (45.8) | 0.252^a^ |
| Median age (IQR) | 87 (80-92) | 84 (77-89) | <0.001^b^ | 86 (80-91) | 82 (76-86) | <0.001^b^ |
| **Clinical variables n (%)** |  |  |  |  |  |  |
| Congestive cardiac failure | 139 (15.0) | 922 (11.5) | 0.002^a^ | 96 (15.7) | 766 (9.8) | <0.001^a^ |
| Chronic kidney disease | 464 (50.0) | 3524 (43.8) | <0.001^a^ | 302 (49.5) | 3127 (40.2) | <0.001^a^ |
| Liver disease | 12 (1.3) | 49 (0.6) | 0.016^a^ | 0 (0) | 23 (100) | 0.179 ^a^ |
| Diabetes | 128 (13.8) | 1214 (15.1) | 0.295^a^ | 108 (17.7) | 1108 (14.2) | 0.019^a^ |
| Palliative care or DNAR | 720 (77.6) | 530 (6.6) | <0.001^a^ | 467 (76.6) | 430 (5.5) | <0.001^a^ |
| CA-AKI | 202 (21.8) | 734 (9.1) | <0.001^a^ | 136 (22.3) | 651 (8.4) | <0.001^a^ |
| **Clinical scores** |  |  |  |  |  |  |
| Median MUST (IQR) | 1 (1-3) | 1 (1-2) | <0.001^b^ | 1 (1-3) | 1 (1-2) | <0.001^b^ |
| Median NEWS (IQR) | 2 (1-5) | 1 (0-3) | <0.001^b^ | 2 (1-5) | 1 (0-3) | <0.001^b^ |
| Mean CFS (SD) | 6.0 (+/-1.5) | 4.9 (+/-1.6) | <0.001^c^ | 6.35 (+/-1.6) | 4.8 (+/-1.6) | <0.001^c^ |
| **Outcomes** |  |  |  |  |  |  |
| Median days in-hospital (IQR) | 11 (5-24) | 10 (4-20) | <0.001^b^ | 12 (6-20) | 9 (5-16) | <0.001^b^ |
| Length of stay >30 days (%) | 149 (16.1) | 1042 (13.0) | 0.08^a^ | 63 (10.3) | 519 (6.7) | 0.001 |
| 30-day readmission (%) | N/A | 1172 (22.0) | N/A | N/A | 1679 (21.6) | N/A |

# Appendix 5: Co-linearity analysis

**Table.** Co-linearity analysis

|  | Tolerance | Variance inflation factor (VIF)^a^ |
| --- | --- | --- |
| Age | 0.889 | 1.124 |
| Frailty | 0.873 | 1.146 |
| MUST | 0.955 | 1.048 |
| NEWS | 0.972 | 1.028 |

^a^ VIF>2.50 used to indicate possible collinearity

# Appendix 6: OPERA risk groups and operating performance measures

**Table.** Risk groups for OPERA, with sensitivity, specificity, positive predictive value (PPV), and negative predictive value (NPV) for mortality. Odds ratio (OR) for extended length of stay (LOS) for each risk group compared to low risk group with (95% confidence intervals).

|  | Risk | Sensitivity (%) | Specificity (%) | PPV (%) | NPV (%) | Mortality in group (%) | Patients in group | Average length of stay (days) | OR: LOS >30 days | OR: LOS >7 days | OR: LOS >2 days |
| --- | --- | --- | --- | --- | --- | --- | --- | --- | --- | --- | --- |
| Derivation | Low (<10pts) | 100.0 | 0.0 | 10.3 | N/A | 3 (0.4) | 693 | 7.7 |  |  |  |
|  | Medium (10-16) | 99.7 | 8.6 | 11.2 | 99.6 | 131 (3.9) | 3372 | 12.9 | 3.7 (2.4-5.9) | 2.0 (1.7-2.4) | 1.7 (1.3-2.1) |
|  | High (17-20) | 85.6 | 48.9 | 16.2 | 96.7 | 247 (9.3) | 2655 | 18.2 | 8.0 (4.9-13.1) | 3.6 (3.0-4.3) | 2.8 (2.2-3.7) |
|  | Severe (21+) | 58.9 | 78.8 | 24.3 | 94.3 | 547 (24.3) | 2254 | 18.3 | 9.8 (6.1-16.0) | 4.8 (4.0-5.8) | 3.0 (2.3-4.1) |
|  |  |  |  |  |  |  |  |  |  |  |  |
| Validation | Low (<10pts) | 100.0 | 0.0 | 7.3 | N/A | 8 (0.9) | 880 | 7.8 |  |  |  |
|  | Medium (10-16) | 98.7 | 11.2 | 8.0 | 99.1 | 79 (2.3) | 3391 | 10.3 | 1.8 (1.4-2.3) | 2.0 (1.7-2.3) | 1.6 (1.2-2.2) |
|  | High (17-20) | 85.7 | 53.8 | 12.7 | 97.7 | 163 (7.1) | 2312 | 15.2 | 4.9 (3.0-7.8) | 4.4 (3.7-5.1) | 3.2 (2.1-4.7) |
|  | Severe (21+) | 59.0 | 81.4 | 19.9 | 96.2 | 360 (19.9) | 1808 | 16.2 | 6.7 (5.2-8.7) | 6.8 (5.6-8.2) | 5.6 (4.3-7.3) |
|  | | | | | |  |  |  |  |  |  |

# Appendix 7: Receiver operating curves

## 48-hour mortality


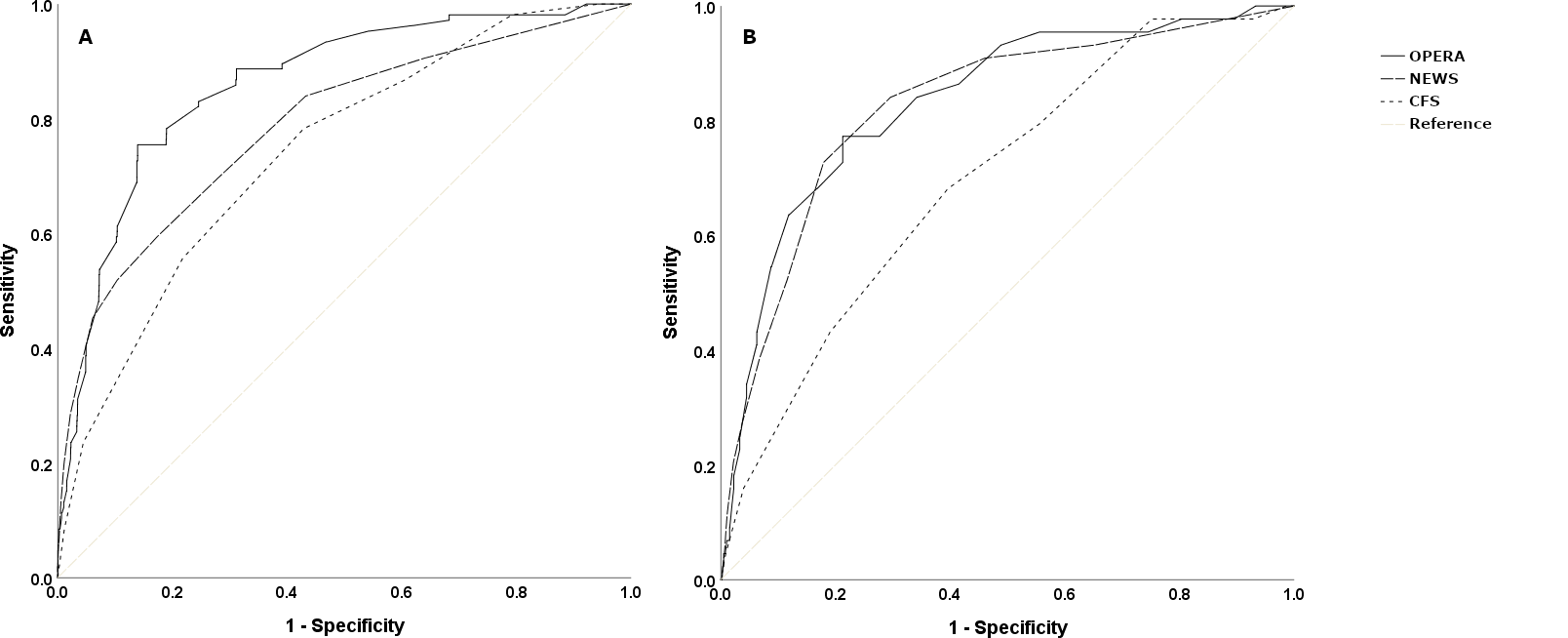


**Figure.** Receiver operating curves for 48-hour mortality for derivation (A) and validation (B) groups. In derivation AUCs: OPERA 0.86 (0.83-0.90), NEWS 0.79 (0.74-0.84), and CFS 0.74 (0.70-0.79). In validation AUCs: OPERA 0.84 (0.78-0.90), NEWS 0.83 (0.76-0.89), CFS 0.70 (0.62-0.77).

## 7-day mortality


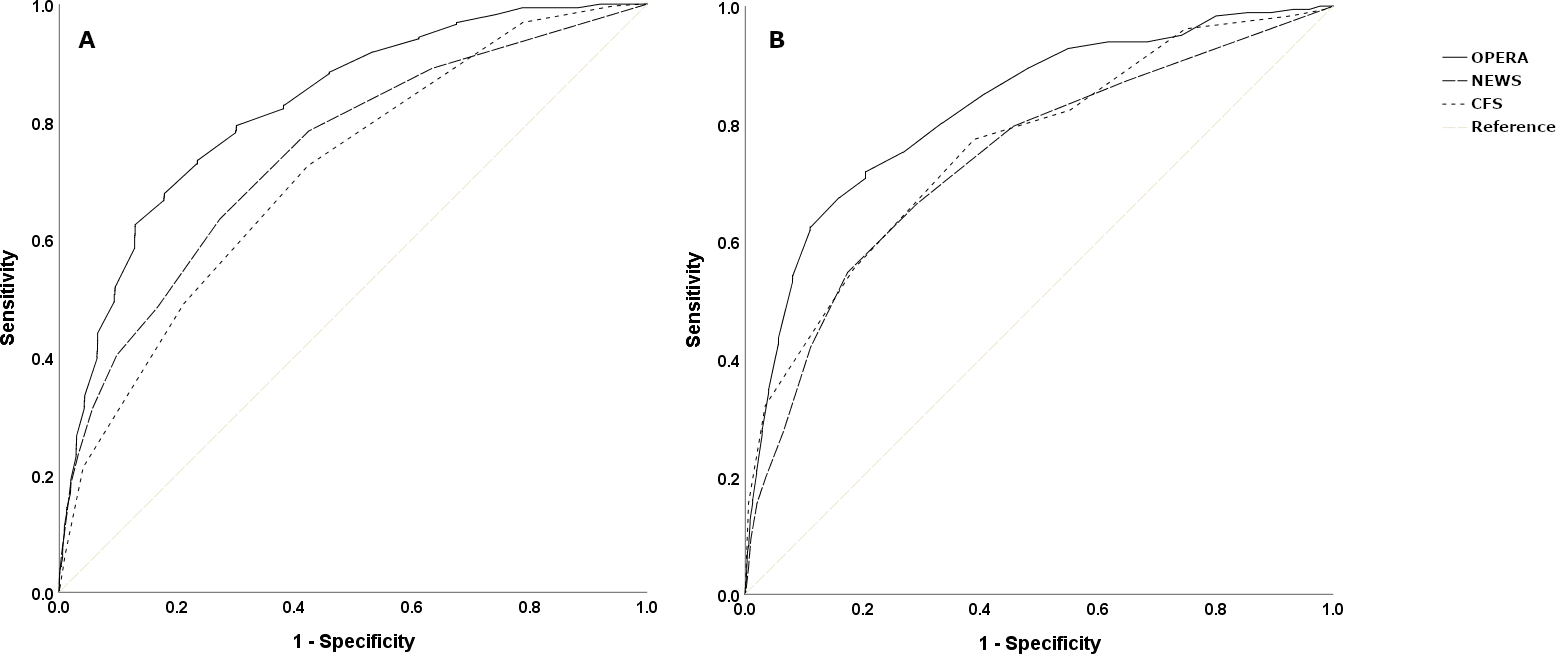


**Figure.** Receiver operating curves for 7-day mortality for derivation (A) and validation (B) groups. In derivation AUCs: OPERA 0.83 (0.81-0.85), NEWS 0.75 (0.72-0.77), CFS 0.71 (0.68-0.74). In validation AUCs: OPERA 0.83 (0.79-0.86), NEWS 0.74 (0.70-0.78, CFS 0.76 (0.72-0.80).

## Length of stay >30 days

## 30-day readmission


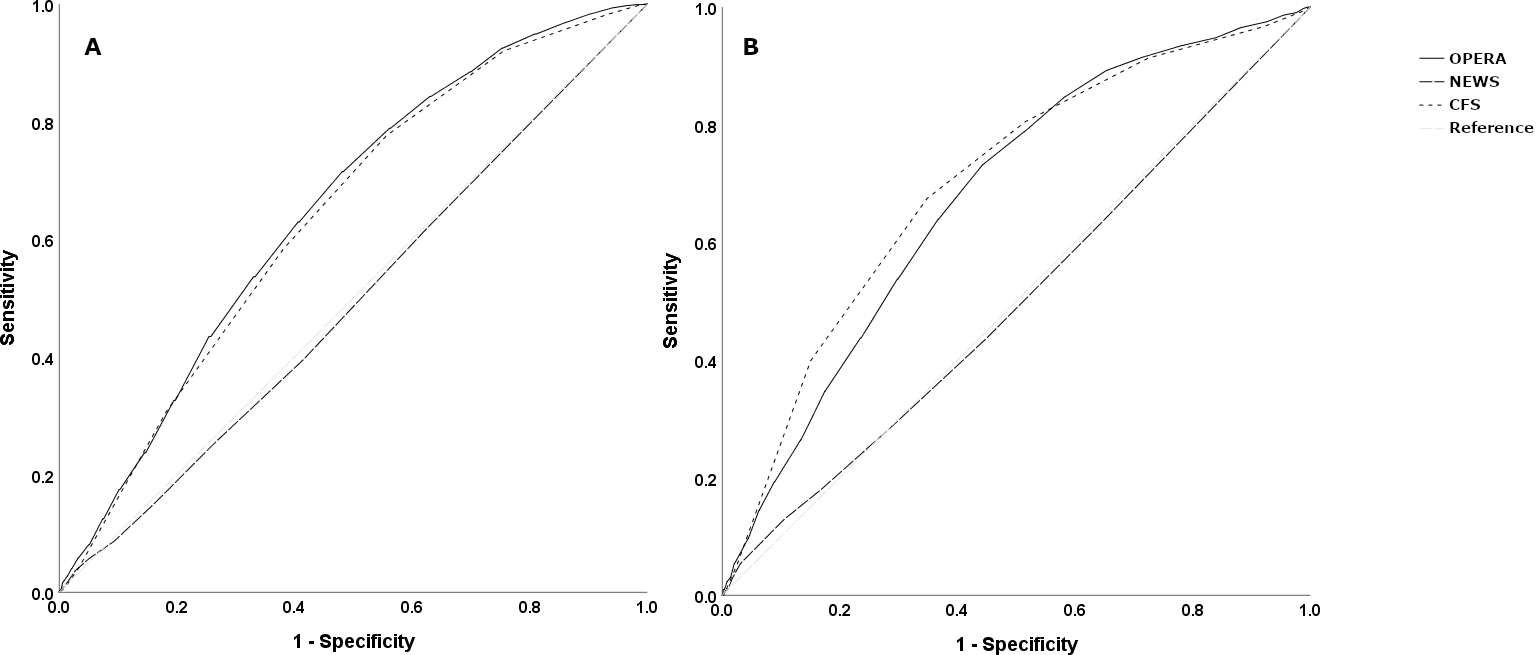


**Figure.** Receiver operating curves for length of stay >30 days for derivation (A) and validation (B) groups. In derivation AUCS: OPERA 0.66 (0.64-0.67), NEWS 0.49 (0.47-0.51), CFS 0.64 (0.62-0.66). In validation AUCs: OPERA 0.68 (0.65-0.70), NEWS 0.50 (0.47-0.52), CFS 0.70 (0.68-0.72).


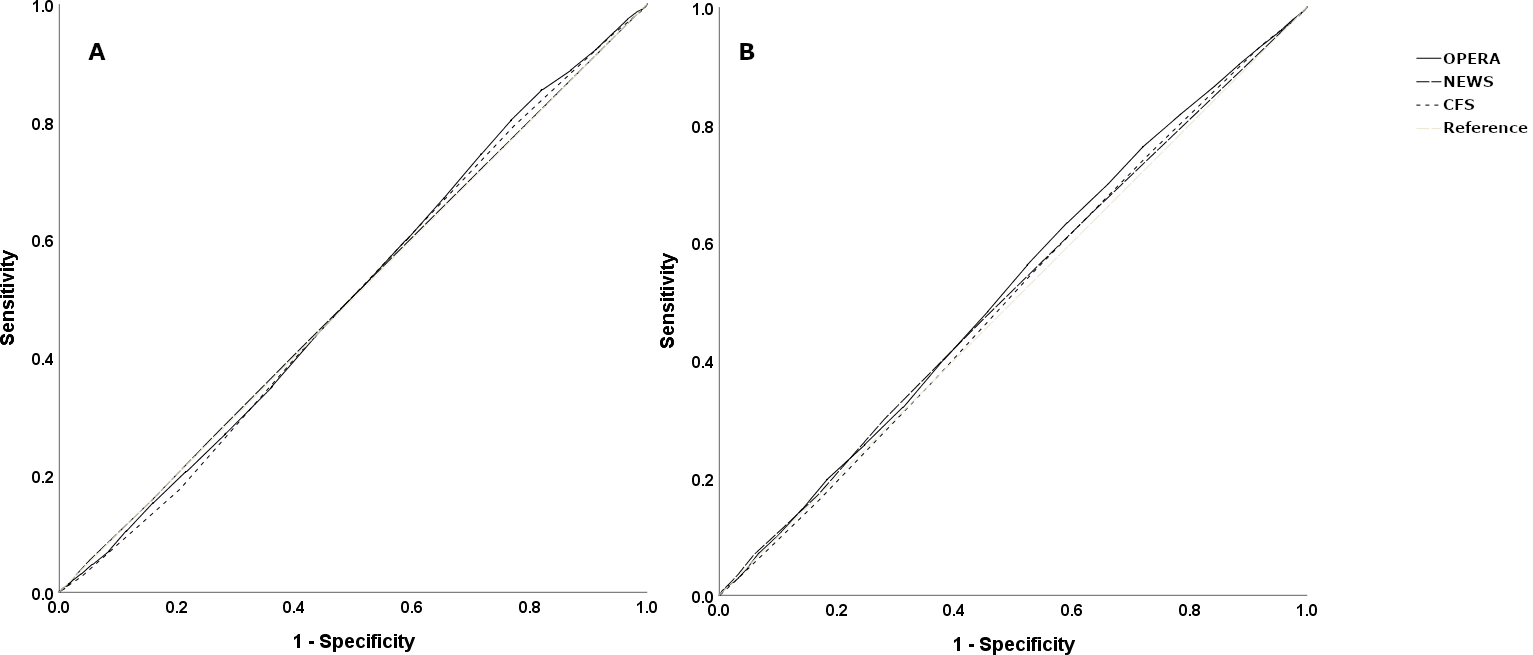


**Figure.** Receiver operating curves for 30-day re-admission for derivation (A) and validation (B) groups. In derivation AUCs: OPERA 0.50 (0.49-0.52), NEWS 0.50 (0.49-0.52), CFS 0.50 (0.48-0.51). In validation AUCs: OPERA 0.52 (0.50-0.54), NEWS 0.51 (0.50-0.53), CFS 0.51 (0.49-0.52).

# Appendix 8: Worked example using the derived regression equation

A worked example of an acutely admitted 85-year-old male with a CFS of 7, AKI, high risk on MUST, and a NEWS of 9. This mortality estimate is obtained from the regression model, prior to its transformation into the points model.

$$Mortality risk= \frac{1}{1+e^{-\left( -8.452+0.809\left( CAAKI \right)+0.345\left( CFS \right)+0.284\left( MUST \right)+0.229\left( NEWS \right)+0.321\left( Male sex \right)+0.040(Age) \right)}}$$

$$Mortality risk= \frac{1}{1+e^{-\left( -8.452+0.809\left( 1 \right)+0.345\left( 7 \right)+0.284\left( 2 \right)+0.229\left( 9 \right)+0.321\left( 1 \right)+0.040(85) \right)}}$$

$Mortality risk=0.754 or 75.4\%$

# Appendix 9: Graphical representation for in-hospital mortality.

**Figure.** Graphical representation of OPERA points vs. in-hospital mortality risk (derivation cohort) (%).

# Appendix 10: Calibration plots using the OPERA regression model.

## Calibration plot for OPERA – regression co-efficient model.


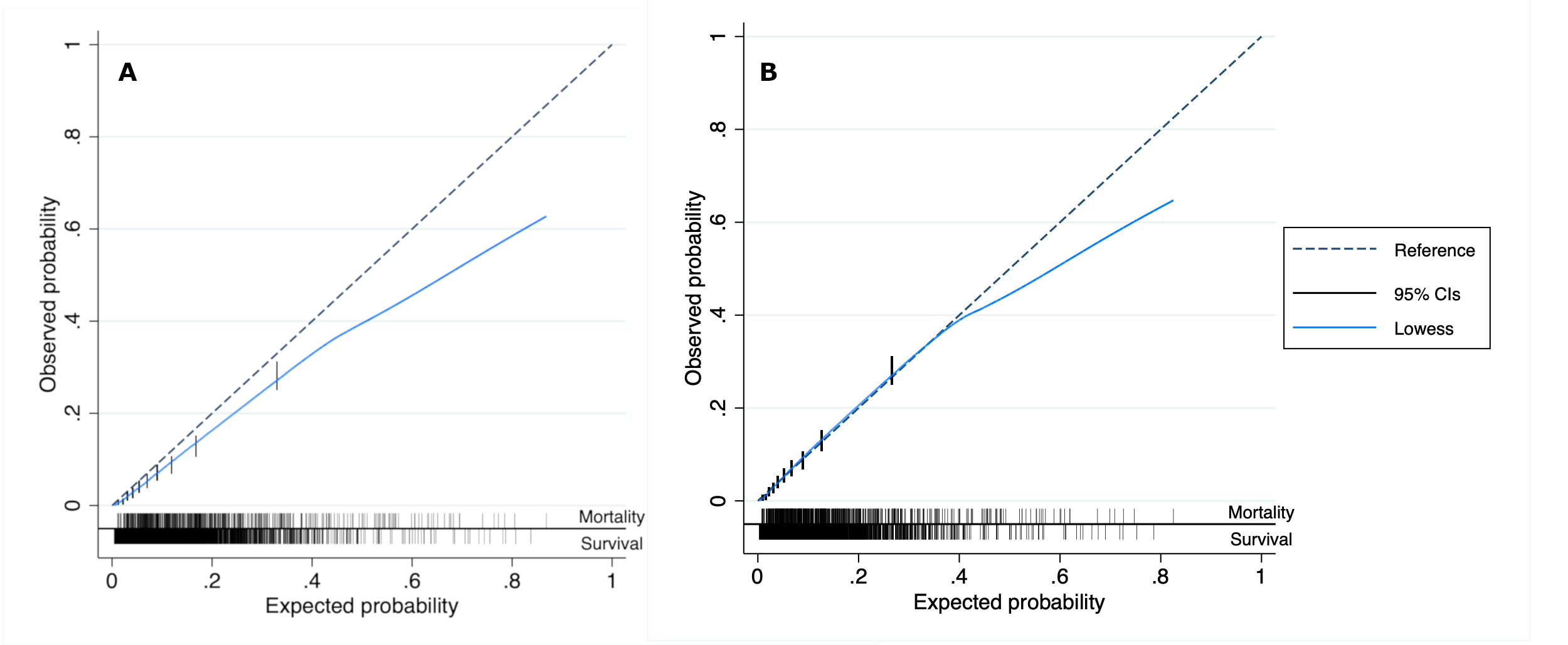


**Figure.** Calibration curve of OPERA using the regression coefficients before recalibration (A) and after recalibration (B). Reference line, 95% confidence interval, lowess smoothing curve, and the distribution of mortality and survival against predicted probabilities

## Recalibrated regression equation and points model equation

$$Mortality risk= \frac{1}{1+e^{-\left( -8.452\mathbf{-0.310}+0.809\left( CAAKI \right)+0.345\left( CFS \right)+0.284\left( MUST \right)+0.229\left( NEWS \right)+0.321\left( Male sex \right)+0.040(Age) \right)}}$$

## Recalibrated points equation.

*Mortality* = $\left. \frac{1}{1+e^{-(-8.452-\boldsymbol{0.310}+65\left( 0.04 \right)+ 0.2\left( \boldsymbol{35} \right))}} \right.$

# Appendix 11: NEWS & CFS missing data analysis

**Table.** Missing value analysis for clinical frailty score (CFS) and NEWS

|  | **Derivation** | | | | | **Validation** | | | | |
| --- | --- | --- | --- | --- | --- | --- | --- | --- | --- | --- |
|  | n/14274 | Death | Survival | Odds ratio for mortality^a^ | P value | n/14079 | Death | Survival | Odds ratio for mortality^a^ | P value |
| Both NEWS & CFS missing | 217 | 26 | 191 | 1.57 (1.03-2.37) | 0.034 | 183 | 28 | 155 | 3.00 (1.99-4.51) | <0.001 |
| Either NEWS or CFS missing | 5083 | 197 | 4886 | 0.35 (0.30-0.41) | <0.001 | 6375 | 229 | 6146 | 0.46 (0.40-0.54) | <0.001 |
| Only missing NEWS | 225 | 37 | 255 | 1.97 (1.38-2.80) | <0.001 | 152 | 15 | 137 | 1.80 (1.05-3.09) | 0.031 |
| Only missing CFS | 4828 | 160 | 4668 | 0.29 (0.25-0.35) | <0.001 | 6224 | 214 | 6010 | 0.44 (0.38-0.52) | <0.001 |
| Neither NEWS or CFS missing | 8972 | 928 | 8044 | 2.63 (2.26-3.05) | <0.001 | 8388 | 610 | 7778 | 1.92 (1.66-2.33) | <0.001 |
| ^a^ In-hospital mortality | | | | | | | | | | |

# Citations used in appendix

**1**. Rockwood K. A global clinical measure of fitness and frailty in elderly people. Can Med Assoc J 2005; 173: 489–495.

**2**. Elia M. Development and use of the ‘Malnutrition Universal Screening Tool’ (‘MUST’) for adults. British Association for Parenteral and Enteral Nutrition 2003 https://www.bapen.org.uk/pdfs/must/must-report.pdf.

**3**. National Early Warning Score (NEWS) 2: Standardising the assessment of acute-illness severity in the NHS. Royal College of Physicians 2017 https://www.rcplondon.ac.uk/projects/outputs/national-early-warning-score-news-2.

**4**. Kidney Disease: Improving Global Outcomes (KDIGO) Acute Kidney Injury Work Group. KDIGO Clinical Practice Guideline for Acute Kidney Injury. Kidney Int 2012; 2: 1–136.

**5**. Algorithm for detecting Acute Kidney Injury (AKI) based on serum creatinine changes with time. Secondary Algorithm for detecting Acute Kidney Injury (AKI) based on serum creatinine changes with time. NHS England 2014 https://www.england.nhs.uk/akiprogramme/aki-algorithm/.
